# Delayed Foreign Body Granuloma Following Sinus Augmentation With Deproteinized Bovine Bone: A Case Report

**DOI:** 10.1155/crid/5283720

**Published:** 2026-04-30

**Authors:** Yaniv Mayer, Yoni Friedlander, Omri Emodi

**Affiliations:** ^1^ Ruth and Bruce Rappaport Faculty of Medicine, Technion – Israel Institute of Technology, Haifa, Israel, technion.ac.il; ^2^ Department of Periodontology, Rambam Health Care Campus, Haifa, Israel, rambam.org.il; ^3^ Department of Oral and Maxillofacial Surgery, Rambam Health Care Campus, Haifa, Israel, rambam.org.il

**Keywords:** case report, deproteinized bovine bone mineral, foreign body granuloma, lateral sinus lift, sinus augmentation

## Abstract

This report describes an uncommon granulomatous foreign body reaction that developed after lateral sinus augmentation with deproteinized bovine bone mineral (DBBM). The patient presented 5 months postoperatively with an extraoral draining fistula and intraoral swelling that failed to resolve with multiple courses of antibiotics and was initially misdiagnosed as a chronic infection. Early surgical exploration with subsequent histopathological analysis identified multinucleated giant cells enclosing residual graft particles, establishing a diagnosis of a noninfectious foreign body granuloma. Following thorough debridement and a 12‐month recovery period, implant placement was carried out successfully, with clinically stable results maintained at 30 months of follow‐up. This case illustrates the importance of considering foreign body reactions when evaluating persistent postoperative complications after sinus augmentation and emphasizes the essential role of tissue biopsy in reaching an accurate diagnosis.

## 1. Introduction

Restoring the edentulous posterior maxilla with dental implants is frequently complicated by insufficient vertical bone height, a consequence of alveolar resorption and progressive sinus pneumatization. To overcome this anatomical limitation, Tatum [1] first described the lateral window sinus floor elevation technique in 1986, which has since become a standard preprosthetic surgical procedure [2]. Accumulated evidence from multiple clinical studies supports the reliability of this approach, with reported implant survival rates consistently exceeding 95% [3–6].

A range of bone substitute materials may be used to fill the augmented sinus space, among them xenografts, allografts, and synthetic alloplastic grafts [7–9]. Deproteinized bovine bone mineral (DBBM) is one of the most frequently selected options owing to its favorable osteoconductive properties and gradual resorption rate, which together facilitate predictable new bone formation within the grafted sinus [10].

While sinus augmentation is generally considered a predictable procedure, adverse events may still occur and are typically classified according to their timing as intraoperative, early postoperative, or late postoperative. Among the more commonly reported intraoperative events are Schneiderian membrane tears, hemorrhage, wound infection, displacement of graft material, and benign paroxysmal positional vertigo [11]. Late or chronic complications, though encountered less often, present distinct challenges in both recognition and management [11].

One particularly infrequent late complication is a granulomatous foreign body response directed against the graft material [12]. This process is initiated when tissue macrophages are unable to completely resorb the implanted particles, prompting their fusion into multinucleated giant cells that encapsulate the residual material. While such responses have been described in association with various implantable medical devices, reports following sinus grafting with DBBM are exceedingly scarce, given the extensive evidence supporting its biological compatibility.

Herein, we present an atypical case of a delayed granulomatous foreign body reaction occurring after lateral sinus augmentation with DBBM. By documenting the clinical course, diagnostic pathway, and successful management of this complication, we aim to alert practitioners to the possibility of noninfectious graft‐related reactions even when well‐established biomaterials are employed. Recognizing this entity in the differential diagnosis of persistent postoperative symptoms is essential for timely intervention and optimal patient outcomes.

## 2. Case Presentation

### 2.1. Patient Information

A 74‐year‐old female, with no significant medical history, presented for periodontal treatment and planned implant rehabilitation in both the posterior maxilla and mandible. Examination revealed Periodontitis Stage IV Grade C and hyperkeratotic buccal mucosa. Biopsy of the mucosa confirmed oral lichen planus. The patient completed Step I and Step II periodontal therapy following the 2018 EFP guidelines and reached optimal periodontal health and a caries‐free status on reevaluation.

### 2.2. Surgical Procedure

**Figure 1:**
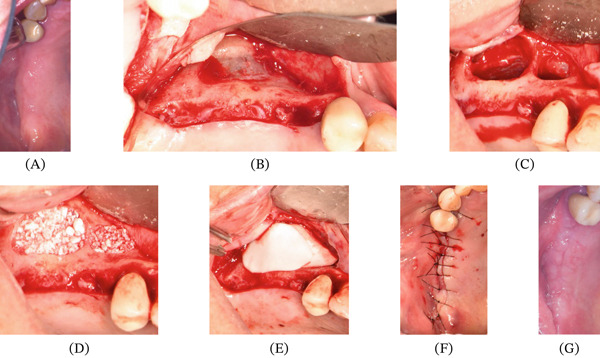
Surgical procedure of lateral sinus lift. (A) Alveolar ridge before. (B) Flap elevation. (C) Lateral window preparation. (D) Graft placement. (E) Collagen membrane placement. (F) Suturing. (G) Two weeks postsurgery.

A lateral window sinus lift was planned for implant placement in the right posterior maxilla (Figure 1A–G). The patient received a single preoperative oral dose of amoxicillin 2 g and dexamethasone 8 mg. Under local anesthesia with lidocaine 2% with epinephrine (1:100,000), a full‐thickness flap was raised from Tooth 14 to the right tuberosity, and a vertical releasing incision was made near the tuberosity. After exposing the lateral wall, a lateral window was created to access the Schneiderian membrane. A bony sinus septum required the creation of a second window for complete membrane elevation. The membrane was elevated without perforation, and the site was augmented using DBBM (Geistlich Bio‐Oss, Geistlich Pharma AG, Wolhusen, Switzerland). Both windows were covered with a resorbable collagen membrane. Primary closure was achieved using 5‐0 nylon sutures (Ethicon, Inc., Somerville, New Jersey, United States). Postoperatively, amoxicillin/clavulanate was prescribed at a total daily dose of 1.5 g, divided into three 500‐mg doses for 7 days, and dexamethasone 8 mg once daily for 2 days.

### 2.3. Postoperative Course

**Figure 2:**
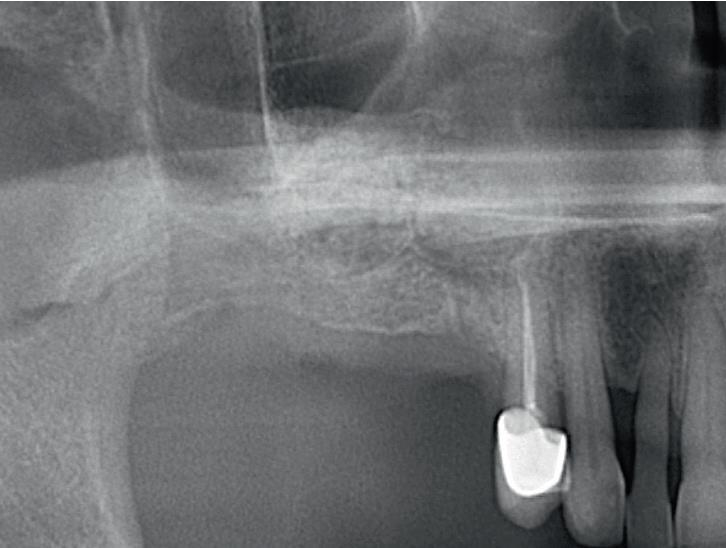
Panoramic radiograph 3 months postsurgery showing no apparent abnormalities.

One week after surgery, the patient developed a rash, presumed to be an allergic reaction to the antibiotic, leading to discontinuation of Augmentin. Healing progressed without further incident. At a 3‐month periodontal maintenance visit, the patient reported a palpable lump in her right cheek. Clinical examination confirmed a mass, and panoramic radiography was unrevealing (Figure 2); ultrasound suggested a probable lipoma. The patient was referred for maxillofacial evaluation but missed the appointment. Given the later identification of DBBM particles within the buccal soft tissues, the clinical findings were interpreted as evidence of displacement of graft material beyond the intended sinus compartment. However, because no cross‐sectional imaging was obtained when the cheek mass was first noted, it was not possible to determine whether this represented early postoperative displacement or later migration.

**Figure 3:**
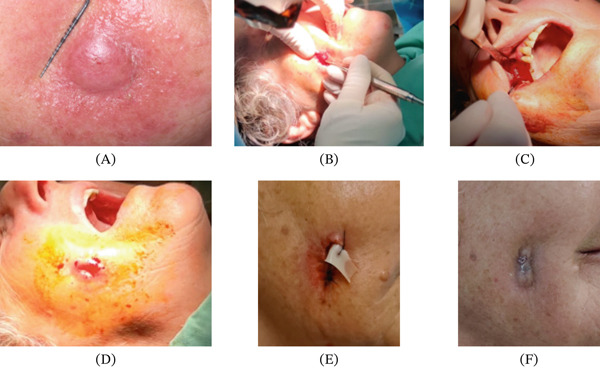
(A) Extraoral suppurative fistula at 5 months postsurgery. (B–D) Surgical curettage procedure showing exploration and debridement. (E, F) Surgical drain placement (3 h).

**Figure 4:**
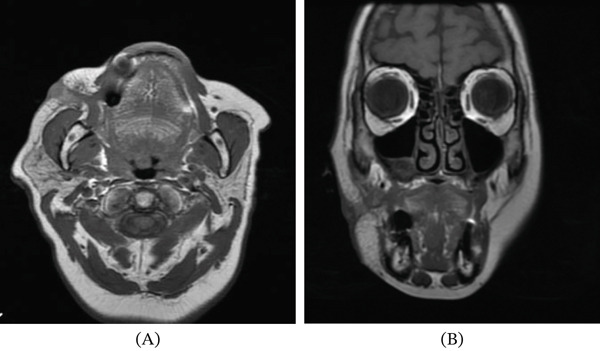
MRI images demonstrating a lesion originating from the right maxillary sinus adjacent to the lateral window sites. (A) Coronal view. (B) Axial view.

**Figure 5:**
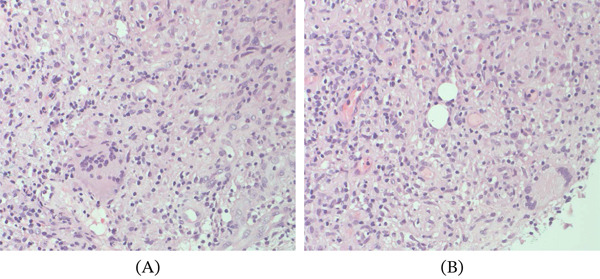
(A, B) Histological examination (H&E staining, ×20) showing multinucleated giant cells, necrotic tissue, and foreign particulate matter.

**Figure 6:**
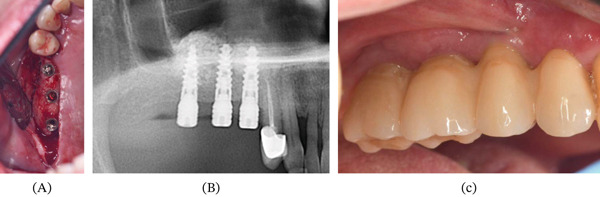
Implants placement 12 months after curettage surgery: (A) clinical view and (B) panoramic radiograph. (C) Final implant‐supported prosthetic rehabilitation at 30 months of follow‐up.

Five months after surgery, the patient returned with an extraoral suppurative fistula and a nodular intraoral mass extending toward the zygoma (Figure 3A). She reported unsuccessful treatment with clindamycin prescribed by a dermatologist. Emergency maxillofacial evaluation suspected a resistant infection. Surgical exploration and curettage revealed remnant xenograft particles in the buccal tissues; curettage and debridement were performed, with placement of a surgical drain (Figure 3B–H). Microbiological cultures were negative for infection. MRI subsequently showed a lesion originating from the right maxillary sinus near the surgical sites (Figure 4A,B). Histopathology revealed necrotic tissue, multinucleated giant cells, and foreign material (Figure 5A,B), leading to a diagnosis of a granulomatous foreign body reaction. The patient underwent a 12‐month healing period before implant rehabilitation was completed, with stable outcomes at 30 months posttreatment (Figure 6A–C).

## 3. Results

Following the diagnosis, implant surgery was postponed for 12 months to allow complete healing and ensure disease resolution. The patient was maintained on a routine periodontal maintenance program with bimonthly monitoring. The surgical site remained stable and asymptomatic throughout the healing period.

After multidisciplinary reassessment involving oral surgery and prosthodontic teams, and radiographic confirmation of complete healing with no anomalies on repeat CT imaging, treatment was resumed. Three implants (Ice, Alpha‐Bio Tec Ltd., Modi’in, Israel) were placed using a surgical guide concurrent with the extraction of Tooth 14, which was deemed prosthetically hopeless. An initial provisional restoration was followed by fabrication of a final cantilever bridge. The patient has remained disease‐free for 30 months posttreatment with successful dental rehabilitation (Figure 6A–C). A chronological summary of the key clinical events, diagnostic tools, and treatment milestones is presented in Table 1. The patient maintained a positive and optimistic attitude throughout the entire course of treatment, expressing full trust in the clinical team. She cooperated willingly with all examinations, diagnostic workups, and treatment instructions, which contributed to the favorable long‐term outcome.

**Table 1:** Timeline of key clinical events and interventions. Summary of major diagnostic, surgical, and follow‐up milestones from the initial sinus lift procedure through final implant rehabilitation.

Time point	Clinical event/intervention	Diagnostic tools used	Details/findings
Day 0	Lateral sinus lift procedure	Preoperative panoramic radiograph, CBCT	Augmentation with DBBM and collagen membranes; no membrane perforation
Week 1	Postoperative follow‐up	Clinical examination	Mild rash attributed to an antibiotic; Augmentin discontinued; healing uneventful
Month 3	Periodontal maintenance visit	Clinical examination, panoramic radiograph, ultrasound	Palpable lump in the right cheek; ultrasound suggested a lipoma; missed referral
Month 5	Return with fistula and swelling	Clinical examination, panoramic radiograph	Extraoral suppurative fistula and intraoral nodule; unresponsive to antibiotics
Month 5	Surgical exploration and biopsy	Intraoperative findings	Removal of residual DBBM particles; microbiological cultures negative
Month 5	Histopathological diagnosis	Light microscopy (H&E staining)	Multinucleated giant cells and foreign material consistent with a granulomatous foreign body reaction
Month 5	Imaging reassessment	MRI	Lesion originating from the right maxillary sinus near the surgical site
Months 6–17	Healing and monitoring	Clinical and radiographic follow‐up	Complete resolution; stable soft tissues; no recurrence
Month 18	Implant placement	Preoperative CBCT and guided surgery planning	Three implants placed (Sites 14–16) using a surgical guide; uneventful healing
Month 30	Final prosthetic rehabilitation	Clinical and radiographic evaluation	Definitive restoration delivered; asymptomatic and stable outcome

## 4. Discussion

Lateral sinus floor elevation continues to serve as a primary strategy for managing the atrophic posterior maxilla, yielding implant survival rates that rival those reported for implants placed in native bone [11]. Nevertheless, the procedure carries a recognized spectrum of risks, ranging from Schneiderian membrane perforation and intraoperative bleeding to postoperative infection, graft particle displacement, and occasionally chronic sinus inflammation or positional vertigo [13, 14]. Whereas the majority of these events manifest during or shortly after surgery, delayed complications are both rarer and more diagnostically challenging. Notably, less invasive alternatives such as the transcrestal mini sinus lift with concurrent grafting and implant insertion have emerged as a potential option for selected patients with severely resorbed maxillae, offering reduced surgical morbidity relative to the conventional lateral approach [15].

Foreign body granuloma formation represents an underrecognized, noninfectious adverse outcome of sinus grafting procedures. The underlying mechanism involves a chronic inflammatory cascade triggered when tissue macrophages fail to degrade the implanted particles; these cells subsequently fuse into multinucleated giant cells that wall off the persistent material. When graft particles migrate beyond the sinus boundaries into surrounding soft tissues, the clinical picture may be misleading, manifesting as unexplained soft tissue swelling or draining cutaneous tracts. Despite extensive documentation of foreign body responses to other classes of implantable devices, such reactions to DBBM are notably rare in light of its long track record of biocompatibility and bone‐conductive capacity.

In the present case, DBBM particles were identified in the buccal soft tissues outside the intended grafted sinus compartment, indicating ectopic displacement of biomaterial. Although the Schneiderian membrane was elevated without visible perforation and the lateral windows were covered with a collagen membrane, the exact timing and pathway of particle displacement cannot be determined retrospectively. Two explanations should therefore be considered: either graft particles were displaced into the buccal tissues at the time of surgery or in the immediate postoperative period and became clinically evident only later, or delayed migration of particles occurred from the grafted area into the adjacent soft tissues. Because no dedicated CT or CBCT was obtained when the initial cheek swelling developed, concomitant sinus involvement could not be fully excluded at that stage. Nevertheless, microbiological cultures were negative, histopathology demonstrated multinucleated giant cells surrounding foreign material, and the overall findings supported a foreign body granulomatous reaction rather than a primary infectious process.

Two principal lessons emerge from this experience. First, when a postoperative lesion in the sinus‐grafted region fails to resolve with standard antimicrobial therapy, a noninfectious cause such as a foreign body reaction must be actively pursued rather than assumed to represent treatment‐resistant infection. Second, early biopsy with histopathological evaluation is the most reliable means of distinguishing between infection and a granulomatous reaction, enabling clinicians to proceed with appropriate surgical debridement rather than prolonged and ineffective antibiotic courses. Practitioners should particularly bear in mind that persistent fistulae or soft tissue masses developing weeks to months after sinus augmentation warrant prompt tissue sampling to guide further management.

Importantly, the material remaining within the sinus was not sampled histologically during the debridement procedure; therefore, assessment of the sinus after treatment relied on clinical follow‐up and subsequent radiographic evaluation, which showed complete healing and no abnormalities on repeat CT before implant placement.

A limitation of this case is the absence of dedicated cross‐sectional imaging at the first presentation of cheek swelling, which prevents definitive distinction between early ectopic placement and later migration of graft particles and does not allow complete exclusion of concomitant sinus involvement at that time.

## 5. Conclusion

When patients present with nonresolving extraoral fistulae or unexplained soft tissue masses months after sinus floor elevation and empiric antibiotic therapy proves ineffective, clinicians should strongly suspect a foreign body granulomatous reaction. Although rare, this complication warrants inclusion in the differential diagnosis of delayed postaugmentation complications. Timely biopsy combined with targeted imaging enables an accurate diagnosis and allows definitive surgical debridement, preserving the opportunity for subsequent implant rehabilitation. As demonstrated in this case, early recognition and appropriate intervention can lead to fully successful prosthetic outcomes despite this unusual complication.

## Author Contributions

**Yoni Friedlander:** conceptualization, methodology, investigation, data curation, formal analysis, visualization, writing – original draft. **Omri Emodi:** methodology, investigation, writing – review and editing. **Yaniv Mayer:** conceptualization, methodology, investigation, formal analysis, writing – original draft, writing – review and editing, supervision.

## Funding

No funding was received for this manuscript.

## Disclosure

All authors have read and approved the final version of the manuscript. Yaniv Mayer (corresponding author) had full access to all of the data in this study and takes full responsibility for the integrity of the data and the accuracy of the data analysis.

## Ethics Statement

This study is a single anonymized case report and does not constitute a research study involving human subjects as defined by institutional or national regulations. Accordingly, formal ethical approval from an Institutional Review Board (IRB) or Ethics Committee was not required. This exemption is consistent with the policies of Rambam Health Care Campus for case reports that do not involve experimental interventions or identifiable patient data. The report was prepared in accordance with the CARE (CAse REport) guidelines (see the Supporting Information section available here). All clinical photographs were de‐identified and do not reveal the identity of the patient. Written informed consent for publication of this case report and all accompanying images was obtained directly from the patient prior to submission.

## Consent

All patients provided consent for personal data processing, and informed consent was obtained from all individual participants included in the study.

## Conflicts of Interest

The authors declare no conflicts of interest.

## Supporting information


**Supporting Information** Additional supporting information can be found online in the Supporting Information section. This case report was prepared following the CARE guidelines to ensure transparency and accuracy in reporting clinical findings. The completed CARE Checklist is available as supporting information.

## Data Availability

The authors confirm that the data supporting the findings of this study are available within the article. This is a single case report, and all relevant clinical, radiographic, histopathological, and follow‐up data are presented in the manuscript text, figures, and table. Additional raw patient data cannot be shared publicly due to privacy and ethical restrictions in accordance with patient confidentiality requirements.
