# Supplementary material for: Delayed Foreign Body Granuloma Following Sinus Augmentation With Deproteinized Bovine Bone: A Case Report
Source: Case Rep Dent. 2026 Apr 30;2026:5283720. doi: 10.1155/crid/5283720 (PMC13130845; doi:10.1155/crid/5283720)
Supplement: Supplementary file 1 — Supporting Information Additional supporting information can be found online in the Supporting Information section. This case report was prepared following the CARE guidelines to ensure transparency and accuracy in reporting clinical findings. The completed CARE Checklist is available as supporting information. [file CRID-2026-5283720-s001.docx]

**CARE Checklist**

*CAse REport (CARE) Guidelines – 2013 Checklist*

**Manuscript title:** Delayed Foreign Body Granuloma Following Sinus Augmentation with Deproteinized Bovine Bone

**Authors:** Yoni Friedlander, Omri Emodi, Yaniv Mayer

**Corresponding author:** Yaniv Mayer (yaniv.mayer@technion.ac.il)

| **Topic** | **Item** | **Checklist Item Description** | **Reported on (Section / Paragraph)** |
| --- | --- | --- | --- |
| **Title** | 1 | The diagnosis or intervention of primary focus followed by the words “case report” | Title: “Delayed Foreign Body Granuloma Following Sinus Augmentation with Deproteinized Bovine Bone” the words “case report” was appended to the title for compliance |
| **Key Words** | 2 | 2 to 5 key words that identify diagnoses or interventions in this case report, including “case report” | Keywords are appeared |
| **Abstract** | 3a | Introduction – What is unique about this case and what does it add to the scientific literature? | Abstract, sentence 1: rare granulomatous foreign body reaction following lateral sinus lift with DBBM |
|  | 3b | The patient’s main concerns and important clinical findings | Abstract: extraoral fistula, intraoral swelling, unresponsive to antibiotics, multinucleated giant cells on histopathology |
|  | 3c | The primary diagnoses, interventions, and outcomes | Abstract: diagnosis of foreign body reaction; debridement performed; implant rehabilitation successful at 30 months |
|  | 3d | Conclusion – What are the “take-away” lessons? | Abstract, final sentence: importance of including foreign body reactions in differential diagnosis; role of histopathology |
| **Introduction** | 4 | Briefly summarizes why this case is unique and may include medical literature references | Introduction section, paragraphs 1–4: describes rarity of granulomatous foreign body reaction to DBBM after sinus lift; references [1–12] |
| **Patient Information** | 5a | De-identified patient specific information | Case Presentation, Patient Information: 74-year-old female |
|  | 5b | Primary concerns and symptoms of the patient | Case Presentation, Patient Information: presented for periodontal treatment and planned implant rehabilitation in posterior maxilla and mandible |
|  | 5c | Medical, family, and psychosocial history including relevant genetic information | Case Presentation, Patient Information: no significant medical history; generalized stage 4, grade C periodontitis; oral lichen planus confirmed by biopsy |
|  | 5d | Relevant past interventions and their outcomes | Case Presentation, Patient Information: completed Step I and Step II periodontal therapy per 2018 EFP guidelines; achieved optimal periodontal health |
| **Clinical Findings** | 6 | Describe significant physical examination (PE) and important clinical findings | Case Presentation, Patient Information: generalized stage 4 grade C periodontitis; hyperkeratotic buccal mucosa; biopsy-confirmed oral lichen planus |
| **Timeline** | 7 | Historical and current information from this episode of care organized as a timeline (figure or table) | Table 1: Timeline of key clinical events and interventions (Day 0 through Month 30) |
| **Diagnostic Assessment** | 8a | Diagnostic methods (PE, laboratory testing, imaging, surveys) | Case Presentation, Postoperative Course: panoramic radiography, ultrasound, MRI, microbiological cultures, histopathological examination (H&E staining) |
|  | 8b | Diagnostic challenges | Case Presentation, Postoperative Course: initial misdiagnosis as lipoma (ultrasound); subsequently misinterpreted as resistant infection; unresponsive to clindamycin and Augmentin |
|  | 8c | Diagnosis (including other diagnoses considered) | Case Presentation, Postoperative Course: final diagnosis – granulomatous foreign body reaction; other diagnoses considered: lipoma, chronic infection |
|  | 8d | Prognostic characteristics when applicable | Results section: favorable prognosis after debridement and 12-month healing period |
| **Therapeutic Intervention** | 9a | Types of therapeutic intervention (pharmacologic, surgical, preventive) | Case Presentation, Surgical Procedure and Postoperative Course: lateral sinus lift with DBBM graft; surgical exploration, curettage, and debridement; pharmacologic (amoxicillin, dexamethasone, amoxicillin/clavulanate, clindamycin); implant placement |
|  | 9b | Administration of therapeutic intervention (dosage, strength, duration) | Case Presentation, Surgical Procedure: amoxicillin 2 g single preoperative dose; dexamethasone 8 mg; amoxicillin/clavulanate 1.5 g/day (3 × 500 mg) for 7 days; dexamethasone 8 mg × 2 days; lidocaine 2% with epinephrine 1:100,000 |
|  | 9c | Changes in therapeutic interventions with explanations | Case Presentation, Postoperative Course: Augmentin discontinued at 1 week due to allergic rash; clindamycin prescribed by dermatologist was unsuccessful; treatment shifted from antibiotic therapy to surgical exploration and debridement once foreign body reaction was diagnosed |
| **Follow-up and Outcomes** | 10a | Clinician- and patient-assessed outcomes (if available) | Results section: surgical site remained stable and asymptomatic; complete healing confirmed by repeat CT imaging; successful implant rehabilitation at 30 months |
|  | 10b | Important follow-up diagnostic and other test results | Results section: radiographic confirmation of complete healing with no anomalies on repeat CT imaging; Figure 6a–c |
|  | 10c | Intervention adherence and tolerability (How was this assessed?) | Results section: patient maintained on routine periodontal maintenance with bimonthly monitoring; disease-free for 30 months |
|  | 10d | Adverse and unanticipated events | Case Presentation, Postoperative Course: allergic rash to Augmentin at 1 week; palpable cheek lump at 3 months; extraoral suppurative fistula and intraoral nodular mass at 5 months; granulomatous foreign body reaction (unanticipated complication) |
| **Discussion** | 11a | Strengths and limitations in your approach to this case | Discussion section: strengths – histopathological confirmation, long-term follow-up (30 months), successful rehabilitation; limitations – single case, delayed initial diagnosis, missed referral appointment |
|  | 11b | Discussion of the relevant medical literature | Discussion section, paragraphs 1–3: review of sinus lift complications, foreign body reactions, DBBM biocompatibility; references [11, 13–15] |
|  | 11c | The rationale for conclusions (including assessment of possible causes) | Discussion section, paragraph 3: diagnostic vigilance paramount; histopathological analysis indispensable for excluding infection and guiding management |
|  | 11d | The primary “take-away” lessons from this case report (without references) in a one paragraph conclusion | Conclusion section: maintain high index of suspicion for foreign body reactions when antibiotic therapy fails; prompt diagnosis through imaging and histopathology enables successful rehabilitation |
| **Patient Perspective** | 12 | The patient should share their perspective on the treatment(s) they received | Included at the end of results |
| **Informed Consent** | 13 | Did the patient give informed consent? Please provide if requested | Ethics Statement: written informed consent for publication of this case report and accompanying images was obtained from the patient |
